# Alcohol-induced tubulin post-translational modifications directly alter hepatic protein trafficking

**DOI:** 10.1097/HC9.0000000000000103

**Published:** 2023-03-24

**Authors:** Raghabendra Adhikari, Ramyajit Mitra, Robert G. Bennett, Benita L. McVicker, Pamela L. Tuma

**Affiliations:** 1 Department of Biology, The Catholic University of America, Washington, District of Columbia, USA; 2 Research Service, VA Nebraska-Western Iowa Health Care System, Omaha, Nebraska, USA; 3 Department of Internal Medicine, University of Nebraska Medical Center, Omaha, Nebraska, USA; 4 Department of Biochemistry and Molecular Biology, University of Nebraska Medical Center, Omaha, Nebraska, USA

## Abstract

Chronic ethanol exposure leads to enhanced protein acetylation and acetaldehyde adduction. Of the multitude of proteins that are modified on ethanol administration, tubulin is among the best studied. However, an open question is whether these modifications are observed in patient samples. Both modifications have also been implicated in promoting alcohol-induced defects in protein trafficking, but whether they do so directly is also unanswered.

**Methods and Results:**

We first confirmed that tubulin was hyperacetylated and acetaldehyde-adducted in the livers from ethanol-exposed individuals to a similar extent as observed in the livers from ethanol-fed animals and hepatic cells. Livers from individuals with nonalcohol-associated fatty liver showed modest increases in tubulin acetylation, whereas nonalcohol-associated fibrotic human and mouse livers showed virtually no tubulin modifications. We also asked whether tubulin acetylation or acetaldehyde adduction can directly explain the known alcohol-induced defects in protein trafficking. Acetylation was induced by overexpressing the α-tubulin–specific acetyltransferase, αTAT1, whereas adduction was induced by directly adding acetaldehyde to cells. Both αTAT1 overexpression and acetaldehyde treatment significantly impaired plus-end (secretion) and minus-end (transcytosis)–directed microtubule-dependent trafficking and clathrin-mediated endocytosis. Each modification led to similar levels of impairment as observed in ethanol-treated cells. The levels of impairment by either modification showed no dose dependence or no additive effects suggesting that substoichiometric tubulin modifications lead to altered protein trafficking and that lysines are not selectively modified.

**Conclusions:**

These results not only confirm that enhanced tubulin acetylation is observed in human livers but that it is most relevant to alcohol-induced injury. Because these tubulin modifications are associated with altered protein trafficking that alters proper hepatic function, we propose that changing the cellular acetylation levels or scavenging free aldehydes are feasible strategies for treating alcohol-associated liver disease.

## Graphical abstract

**Figure:**
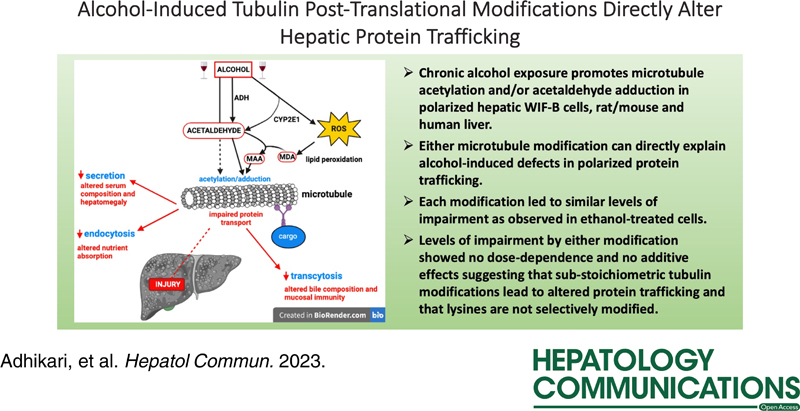

The liver is the major site of ethanol metabolism, making it especially vulnerable to injury from chronic alcohol exposure. Ethanol itself is not hepatotoxic; rather, its metabolites and metabolic byproducts contribute to liver injury.^1^ In hepatocytes, ethanol is oxidized by alcohol dehydrogenase (ADH) and cytochrome P450 2E1 (CYP2E1) to acetaldehyde, which is then metabolized to acetate by aldehyde dehydrogenase. CYP2E1-mediated ethanol metabolism also produces highly reactive oxygen and hydroxyethyl radicals.^1^ These oxygen radicals contribute to enhanced oxidative stress and can promote lipid peroxidation and the production of other reactive metabolites. Many of these reactive intermediates and acetaldehyde readily covalently modify proteins, DNA, and lipids.^2^–^4^ Alcohol exposure also induces post-translational modifications that are common among proteins.^5^–^7^ Of particular interest is alcohol-induced lysine acetylation. To date, ethanol exposure has been shown to induce lysine acetylation of numerous proteins, and that list is expanding.^6^,^7^ The reversibility of acetylation, along with the dozens of lysine deacetylases (KDACs) and lysine acetyltransferases (KATs) that catalyze these reactions strongly suggest that acetylation regulates cellular processes, not unlike phosphorylation.^8^ Thus, one hypothesis for alcohol-induced injury is that the accumulated covalent modifications disrupt proper macromolecular function, thereby, promoting hepatic dysfunction and liver injury.

Dozens of proteins are shown to be covalently lysine modified by acetaldehyde.^9^–^14^ Of these proteins, tubulin adduction is among the best studied. *In vitro*, α-tubulin is preferentially modified on a highly reactive lysine,^15^,^16^ which leads to drastically impaired microtubule polymerization.^17^ This was confirmed in isolated hepatocytes from alcohol-fed rats and WIF-B cells.^18^,^19^ However, we also found that microtubules were acetylated 2- to 3-fold more on lysine 40 of α-tubulin in ethanol-treated cells.^18^ This modification is associated with stable microtubules that are characterized by a longer half-life and resistance to microtubule poisons.^20^ Thus, ethanol adduction impairs tubulin polymerization, but once microtubules are formed, they are hyperacetylated and stabilized.

Over the last several years, we determined that the alcohol-induced defects in both minus- and plus-end–directed microtubule-dependent protein trafficking described decades ago in rodent models and inferred in patients with alcohol-associated liver disease can be explained by increased microtubule acetylation.^21^ We have further shown that increased microtubule acetylation may likely lead to impaired vesicle motility by impeding microtubule-based motor translocation and processivity along the filamentous tracks.^22^,^23^ Our early studies found that increased microtubule acetylation was prevented by 4-methyl pyrazole (an ADH inhibitor) and potentiated by cyanamide (an aldehyde dehydrogenase inhibitor), indicating that ADH-mediated ethanol metabolism was required.^18^ Since then, we have found that the alcohol-induced impairments in protein trafficking are also dependent on ADH-mediated ethanol metabolism, implicating tubulin adduction as another possible explanation for the observed impairments.^21^,^23^–^26^

Our previous studies have been limited to rodent models of alcohol-induced liver disease and hepatic cells in culture. To extend our studies, we importantly confirmed that human liver tissue also exhibits alcohol-induced acetylation and/or adduction. We further asked whether enhanced acetylation and/or adduction are specific to alcohol-induced liver injury. Although our previous studies have strongly correlated the enhanced tubulin acetylation with known alcohol-induced defects in microtubule-dependent protein trafficking and lipid droplet dynamics, we tested this directly. Because microtubules are also targeted for acetaldehyde adduction, we asked whether this modification can also lead to impaired protein trafficking. In addition, because both modifications occur on lysine residues, we further examined whether tubulin acetylation or acetaldehyde adduction alone or in combination can directly explain alcohol-induced defects in protein trafficking. The results presented here have answered these important questions.

## METHODS

### Cell culture

WIF-B cells were grown in a humidified 7% CO_2_ incubator using F12 (Coon) media (Sigma-Aldrich, St. Louis, MO), supplemented with 5% fetal bovine serum (Gemini Bio-Products, Woodland, CA), 10 μM hypoxanthine, 40 nM aminopterin, and 1.6 μM thymidine at pH 7.0.^27^ Cells were seeded on glass coverslips at 1.3×10^4^ cells/cm^2^ and cultured for 7–10 days until they reached maximal polarity. Cells were treated with 50 mM ethanol in a medium buffered with 10 mM HEPES, pH 7.0, for 72 hours^18^,^28^ or with acetaldehyde for 24 hours.^29^

### Animal studies

All animal studies complied with the ARRIVE guidelines and all of the procedures were approved by the Nebraska-Western Iowa Health Care System Institutional Animal Care and Use Committee. Male Wistar rats (175–200 g) (Charles River Laboratories, Portage, MI) were weight-matched and pair-fed Lieber-DeCarli control or ethanol liquid diets (Dyets Inc., Bethlehem, PA) for 6–8 weeks as descibed.^30^ Livers were flash frozen at sacrifice and stored at −70 °C. Male 6-week-old C57BL/6J mice (Jackson Laboratories, Bar Harbor, ME) were randomly assigned to be treated with carbon tetrachloride (CCl_4_) (diluted 1:7 in sunflower oil) and injected intraperitoneally twice per week with a dose of 1 μL/g body weight (0.125 μL/g CCl_4_) for 4 or 12 weeks (n=6 per group). Control mice received injections of oil alone. Mice had free access to water and chow throughout the study. Mice were euthanized 24 hours after the final injection, and blood and tissues were collected. Formalin-fixed paraffin-embedded sections were stained with hematoxylin and eosin or with picrosirius red to monitor collagen deposition. Images were captured in a blinded fashion using a Nikon Eclipse 80i microscope and DSQilMc digital camera (Boyce Scientific Inc., Gray Summit, MO).

### Human tissue

All research involving human tissue specimens was conducted in accordance with both the Declarations of Helsinki and Istanbul, and the specimens were obtained by written, informed consent. The use of human tissue was approved by the University of Nebraska Medical Center Institutional Review Board (IRB #0155-14-FB). Six normal and four NAFLD human livers were obtained through the Liver Tissue Cell Distribution System, Minneapolis, MN, which was funded by National Institutes of Health contract # HSN276201200017C. All the other human liver samples were donated to research to Live On Nebraska Organ Recovery according to the articles of Authorization for Anatomical Gift or Authorization for Research, Medical Education, and International Use. Alcohol consumption history was confirmed using questionnaires. Tissue was perfused with cold University of Wisconsin organ preservation solution at the time of collection and stored in ice-cold University of Wisconsin solution until transfer to the laboratory. Within 3 hours of collection, formalin-fixed paraffin-embedded sections were prepared. The sections were stained with hematoxylin and eosin using standard techniques. Images were acquired as described above.

### Immunofluorescence microscopy

WIF-B cells were fixed and permeabilized with methanol at −20°C for 5 minutes or were fixed for 1 minute on ice with PBS containing 4% paraformaldehyde and permeabilized for 10 minutes with ice-cold methanol. Cells were processed for indirect immunofluorescence as described^31^ and labeled for the indicated antibodies (Table S1, http://links.lww.com/HC9/A217). To remove soluble tubulin to better visualize the microtubule morphology in acetaldehyde-treated cells, the cells were permeabilized with 0.1% (v/v) Triton X-100, 100 mM PIPES, 1 mM EGTA, 1 mM MgCl_2_ (pH 6.8), and 8% sucrose for 2 minutes before fixing with 4% paraformaldehyde for 30 minutes at room temperature. Cells were visualized using an Olympus BX60 fluorescence microscope (OPELCO, Dulles, VA). Images were acquired with a CoolSnap HQ2 digital camera (Photometrics, Tucson, AZ) using Metamorph image analysis software (Molecular Devices, Sunnyvale, CA).

### Immunoblotting and Immunoprecipitation

WIF-B cells grown on coverslips were washed with chilled PBS, lysed in sample buffer, and boiled for 3 minutes. Excised rat/mouse livers (20% w/v) were Dounce-homogenized in 0.25 M sucrose, 10 mM Tris, and pH 7.4. Human liver pieces (20% w/v) were lysed with Qproteome lysis buffer (Qiagen, Germantown, MD) and homogenized using a BeadBug Homogenizer (Benchmark Scientific Inc., Sayreville, NJ) in tubes containing 3 mm zirconium beads with 3 rounds at 4500 rpm for 50 seconds each at 4 °C. Samples were mixed with sample buffer and boiled for 3 minutes. Proteins were separated using SDS-PAGE, transferred to nitrocellulose, and immunoblotted with the indicated antibodies (Table S1, http://links.lww.com/HC9/A217). Immunoreactivity was detected with enhanced chemiluminescence (PerkinElmer, Crofton, MD). Densitometric analysis of immunoreactive bands was used to calculate relative protein levels using ImageJ software (National Institutes of Health, Bethesda, MD). Lysine-acetylated proteins were recovered from cell lysates (1 mg total protein) using acetyl-lysine affinity beads, according to the manufacturer’s instructions (Cytoskeleton Inc., Denver, CO). The bound samples were collected and immunoblotted to detect global protein and tubulin-specific acetylation.

### Protein trafficking in live cells

To monitor transcytosis, cells were basolaterally labeled with anti-aminopeptidase N (APN) antibodies for 20 minutes at 4°C.^32^ Cells were washed extensively in precooled medium, and 1 set of coverslips was fixed immediately after labeling and represents 0 minutes chase samples. The parallel sets of coverslips were washed, reincubated with the prewarmed medium, and the antibody-antigen complexes were chased to the apical surface for 45 or 90 minutes at 37°C. Cells were processed for immunofluorescence labeling. From micrographs (5–10 nonoverlapping fields per slide), the ratio of canalicular/basolateral fluorescence intensity was determined using ImageJ Measure ROI tool.^22^,^32^ To monitor secretion, cells were rinsed with prewarmed serum-free medium, reincubated in serum-free medium, and media aliquots were collected after 0–60 minutes.^25^ Albumin secretion was detected by immunoblotting. Values were normalized to total cellular α-tubulin levels collected from cell lysates.

### Statistical analysis

The results are expressed as the mean±SEM from at least 3 independent experiments. Comparisons were made using the Student 2-tailed *t* test for paired data. One-way ANOVA was used when more than 2 groups were analyzed, followed by Holm-Sidak multiple pairwise comparison. *p*-values of ≤0.05 were considered significant.

## RESULTS

### Microtubule acetylation and acetaldehyde adduction are highly associated with alcohol-associated liver injury in animal models and humans

**FIGURE 1:**
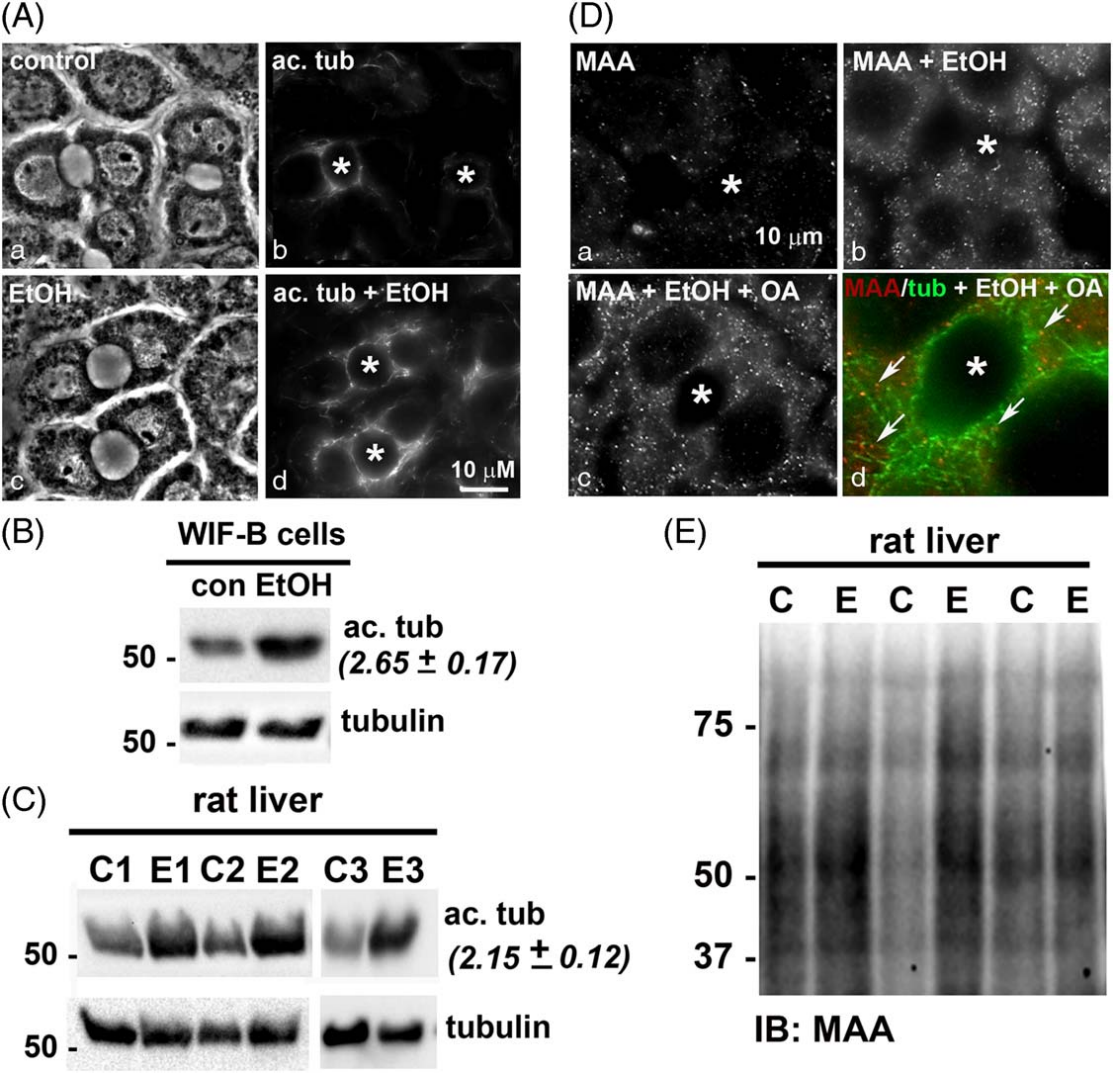
Ethanol exposure induces tubulin acetylation and acetaldehyde adduction. (A) WIF-B cells were treated in the absence or presence of 50 mM ethanol for 72 hours, fixed, and stained for acetylated (ac.) α-tubulin (b and d). Phase images are shown (a and c). Asterisks mark the bile canaliculi. (B) Cell lysates from control (C) or ethanol-treated (EtOH) WIF-B cells were immunoblotted for total or acetylated α-tubulin as indicated. Relative levels of tubulin acetylation were determined by densitometry and normalized to total α-tubulin. The fold-increase in acetylated tubulin in ethanol-treated cells is indicated in parentheses and represents the mean±SEM from at least 3 independent experiments. Molecular weight standards are indicated on the left in kilodalton. (C) Liver homogenates from 3 sets of pair-fed rats (C, control; E, ethanol) were immunoblotted for total or acetylated α-tubulin. The fold-increase in acetylated tubulin is indicated in parentheses and represents the mean±SEM from the 3 pairs of rats. Molecular weight standards are indicated on the left in kilodalton. (D) WIF-B cells were treated in the absence (a) or presence of 50 mM ethanol (b) or ethanol in the additional presence of 200 μM oleic acid (OA) (c and d), fixed and stained for MAA (a-c) or MAA and α-tubulin (merged image in d). Arrows indicate MAA labeling associated with microtubules. Asterisks mark the bile canaliculi. (E) Liver homogenates from three sets of pair-fed rats were immunoblotted for MAA. Molecular weight standards are indicated on the left in kilodalton (*p≤0.05). Abbreviations: MAA, malondialdehyde/acetaldehyde adduct.

Previously, we determined that microtubules are more highly acetylated and more stable in ethanol-treated WIF-B cells, liver slices, and livers from ethanol-fed rats/mice.^18^ We first confirmed the results here. The typical, polarized hepatic morphology of WIF-B cells is shown (Figure 1A). The phase lucent structures formed between 2 cells are analogous to bile canaliculi with tight junctions separating them from the basolateral domain. In control cells, faint labeling of acetylated microtubules was observed emanating from the canalicular surfaces (Figure 1A). In contrast, robust labeling was observed in ethanol-treated cells emanating into the cellular periphery (Figure 1A). Immunoblots of whole lysates confirmed these observations (Figure 1B). When quantitated and normalized to total α-tubulin, acetylated tubulin was detected 2.65-fold over that observed in the control (Figure 1B). Also, as we have shown,^18^ α-tubulin acetylation was enhanced in the livers from ethanol-fed rats. Immunoblots of whole homogenates from 3 sets of pair-fed rat livers showed enhanced acetylated α-tubulin immunoreactivity in samples from ethanol-fed rats (Figure 1C). When quantitated and normalized to total α-tubulin, the acetylated α-tubulin levels were increased 2.15-fold (Figure 1C).

We next asked whether the adduction was observed in WIF-B cells and also confirmed adduction in the livers from ethanol-fed rats. Because acetaldehyde adducts are not stable, we labeled WIF-B cells with antibodies against the combination malondialdehyde/acetaldehyde (MAA) adduct as a readout for acetaldehyde adduction.^33^ In control cells, only background labeling was observed, whereas enhanced MAA staining was observed in ethanol-exposed cells that were more intense in cells also treated with oleic acid to induce lipid accumulation (Figure 1D). In cells additionally labeled for tubulin, many of the MAA-positive puncta colocalized with microtubules consistent with their adduction (Figure 1D, d). As predicted, immunoblots of 3 sets of pair-fed rat samples revealed multiple MAA-reactive species with enhanced immunoreactivity in samples from ethanol-fed rats confirming MAA adduction (Figure 1E).

**FIGURE 2:**
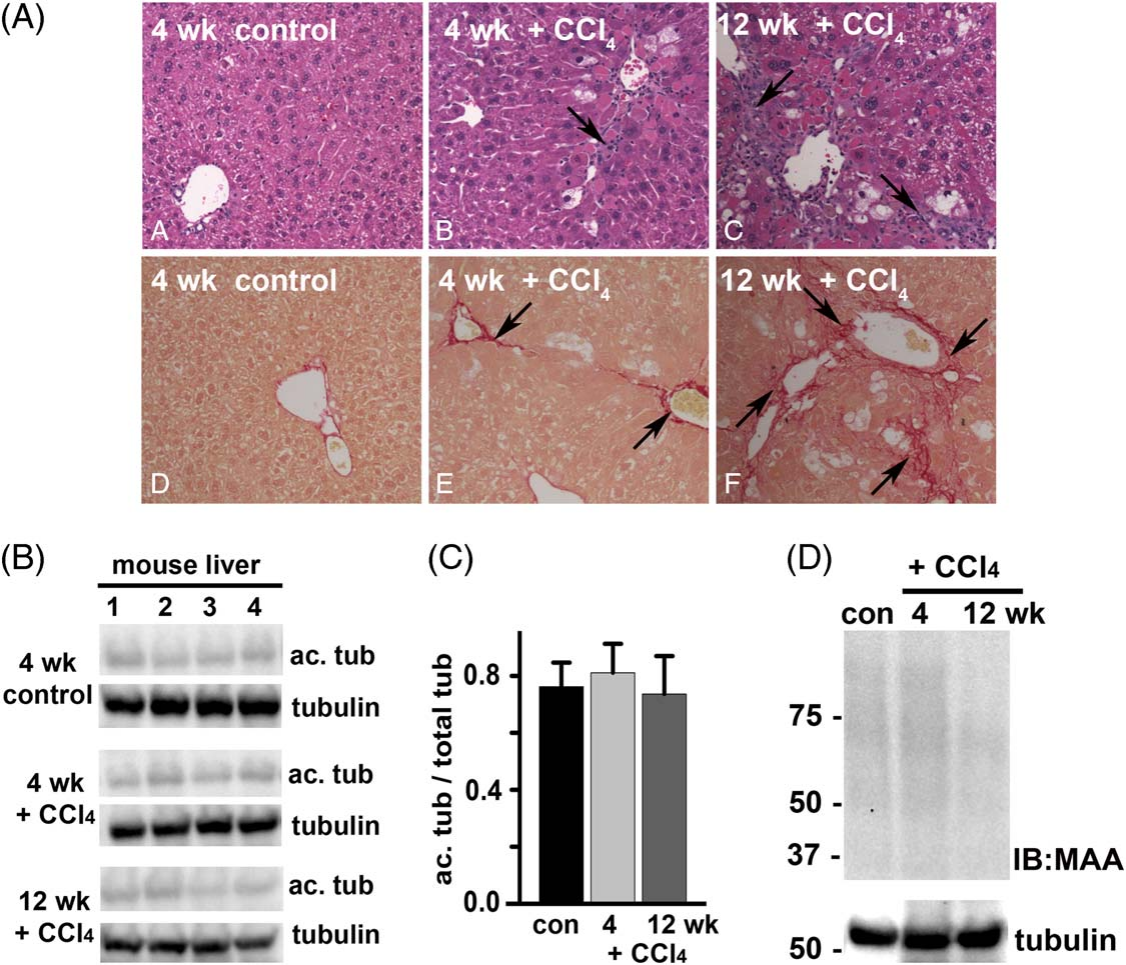
CCl_4_-induced liver injury does not enhance tubulin acetylation or acetaldehyde adduction. (A) Sections from mouse livers treated in the absence (control) or presence of CCl_4_ for 4 or 12 weeks, as indicated, were stained with hematoxylin and eosin using routine procedures (top panels) or were stained with Sirius red to monitor collagen deposition (lower panels). Arrows in the upper panels indicate septa formation and inflammatory cell infiltration. Arrows in the lower panels indicate the regions of collagen deposition. (B) Liver homogenates from control or CCl_4_-treated mice (n=4) were immunoblotted for acetylated and total α-tubulin as indicated. (C) Relative levels of enhanced acetylation were calculated by densitometric analysis of the immunoreactive bands normalized to total α-tubulin. (D) Representative liver homogenates from control or CCl_4_-treated mice were immunoblotted for malondialdehyde/acetaldehyde adduct. Tubulin was used as a loading control. Molecular weight standards are indicated on the left in kilodalton. Abbreviation: CCl_4_, carbon tetrachloride.

To determine whether enhanced tubulin acetylation and adduction were specific to alcohol-induced liver injury, we examined mouse fibrotic livers harvested after 4 or 12 weeks of CCl_4_ exposure. To confirm liver injury in our model, we assessed liver morphology in hematoxylin and eosin–stained tissue sections and confirmed fibrosis using Sirius red staining. The normal hepatic architecture was observed with no collagen deposition in control livers (Figure 2A). Mice treated with CCl_4_ for 4 weeks developed mild fibrosis, where fibrous septa, inflammatory cell infiltration, and areas of necrosis were observed with collagen deposition (Figure 2A). Severe fibrosis was observed in mice treated for 12 weeks, as evident by thicker septa, increased inflammatory cell infiltration, and necrosis with enhanced collagen deposition (Figure 2A). When quantitated, the area of collagen deposition was significantly greater in CCl_4_-treated mice than in the control (Table S2, http://links.lww.com/HC9/A217). Analysis of other markers of liver injury confirmed the morphological observations (Table S2, http://links.lww.com/HC9/A217). Liver homogenate immunoblots from control and treated mice revealed no significant differences in acetylated tubulin; only faint levels were detected (Figure 2B). When quantitated and normalized to total α-tubulin, no significant difference in acetylation was observed (Figure 2C). Similarly, no MAA protein adducts were detected in the livers from control or CCl_4_-treated mice (Figure 2D). Together, these results suggest that protein acetylation and adduction are more highly associated with alcohol-induced injury.

**FIGURE 3:**
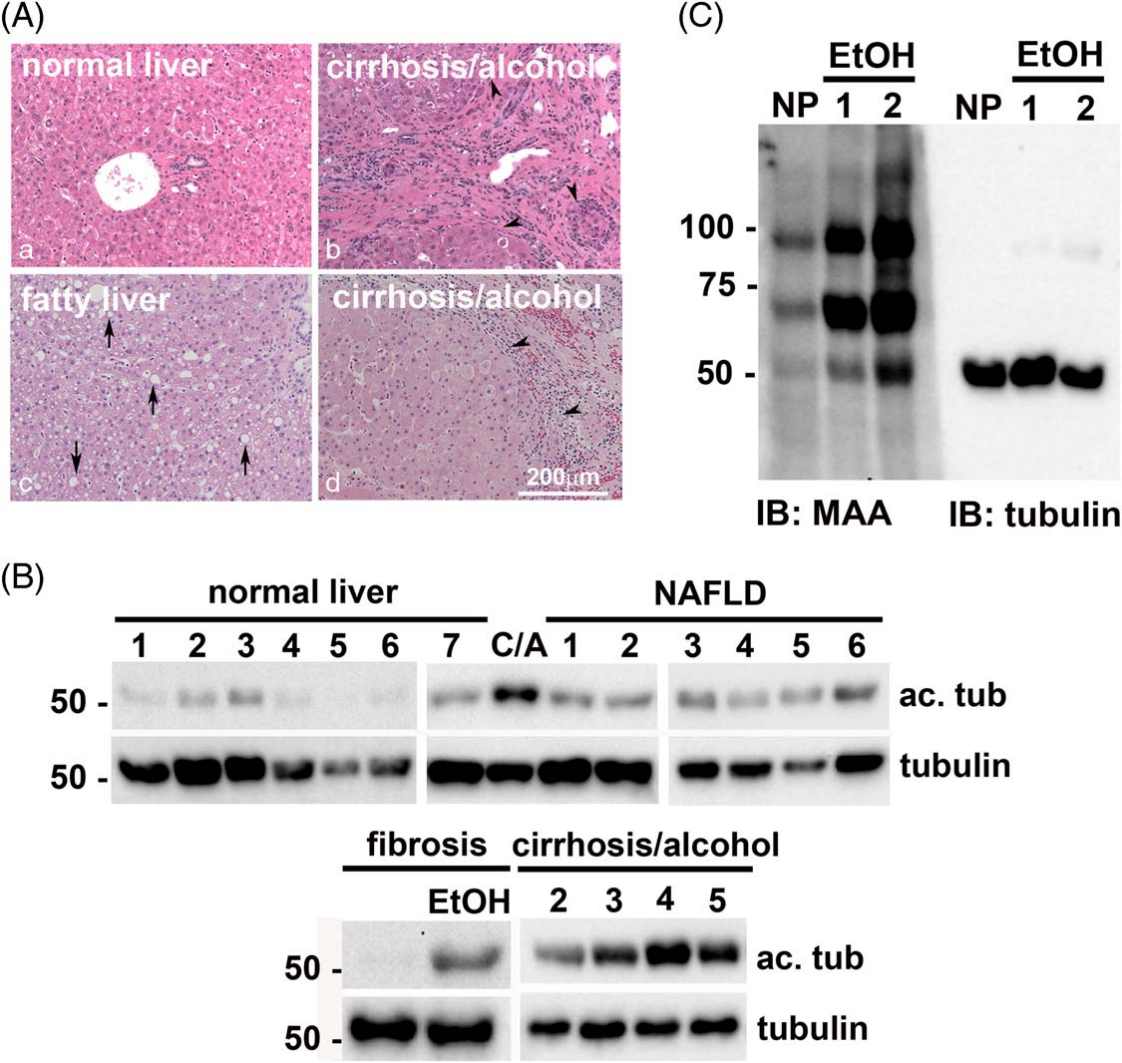
Ethanol exposure selectively enhances tubulin acetylation and acetaldehyde adduction in human livers. (A) Human liver sections with no pathology (a), alcohol-associated cirrhosis (b and d) or NAFLD (c) were stained with hematoxylin and eosin using routine procedures. Arrows indicate lipid droplets; black arrowheads indicate the fibrous layer with immune infiltration and septum formation. (B) Whole homogenates from human livers exhibiting no pathology (normal liver) alcohol-associated cirrhosis (CA) or NAFLD were immunoblotted for acetylated and total α-tubulin as indicated. Molecular weight standards are indicated on the left in kilodalton. (C) Whole homogenates from human livers exhibiting nonalcohol- or alcohol-associated fibrosis and alcohol-associated cirrhosis were immunoblotted for acetylated and total α-tubulin as indicated. Molecular weight standards are indicated on the left in kilodalton. Representative normal and alcohol-exposed human liver homogenates were immunoblotted for MAA protein adducts or total α-tubulin. Molecular weight standards are indicated on the left in kilodalton. Abbreviations: MAA, malondialdehyde/acetaldehyde adduct.

To confirm whether human livers also display alcohol-induced acetylation/adduction, we compared normal human liver samples to samples exhibiting different forms of liver injury. As expected, normal hepatic morphology was observed in the hematoxylin and eosin–stained section of a normal liver (Figure 3A, a), whereas lipid droplets were observed in sections from NAFLD (Figure 3A, c) or alcohol-associated cirrhotic livers (Figure 3A, b and d). Septa formation and immune infiltration were additionally observed in the cirrhotic sections. Analysis of other liver injury markers confirmed the morphological observations (Table S3, http://links.lww.com/HC9/A217). On immunoblots of whole homogenates, only low levels of acetylated tubulin were detected in the normal livers (Figure 3B). NAFLD livers showed slightly enhanced immunoreactivity, whereas samples from alcohol-associated fibrotic and cirrhotic livers displayed much higher immunoreactivity (Figure 3B). Interestingly, the nonalcoholic fibrotic liver showed virtually no labeling, consistent with the results from CCl_4_-treated mice. Signal intensity ratios of acetylated α-tubulin/total α-tubulin were calculated to compare relative tubulin acetylation levels. The ratios from normal livers had similar values (from 0.11 to 0.31) that were somewhat higher in NAFLD livers (from 0.30 to 0.54) and even higher in livers with alcohol-induced injury (from 0.60 to 1.08) (Table 1). When averaged, acetylation was enhanced 2.3- and 4.3-fold over normal in NAFLD livers and livers with alcohol-associated injury, respectively.

**TABLE 1:** Ethanol exposure selectively enhances tubulin acetylation in human livers with alcohol-associated liver injury (samples used for immunoblotting)

Liver pathology	Signal (ac. tub/total)	Liver pathology	Signal (ac. tub/total)	Liver pathology	Signal (ac. tub/total)
Normal 1	0.11	NAFLD 1	0.46	Fibrosis	0.04
Normal 2	0.15	NAFLD 2	0.30	Fibrosis/alcohol incomplete cirrhosis	**0.60**
Normal 3	0.28	NAFLD 3	0.47	Cirrhosis/alcohol 1	**0.74**
Normal 4	0.21	NAFLD 4	0.36	Cirrhosis/alcohol 2	**0.70**
Normal 5	0.08	NAFLD 5	0.46	Cirrhosis/alcohol 3	**0.86**
Normal 6	0.22	NAFLD 6	0.54	Cirrhosis/alcohol 4	**1.08**
Normal 7	0.31			Cirrhosis/alcohol 5	**0.90**

*Note:* Human normal livers 1–6 and NAFLD livers 3–6 were obtained through the Liver Tissue Cell Distribution System, Minneapolis, Minnesota, which was funded by NIH contract # HSN276201200017C. All other livers were from the Live On Nebraska organ recovery program. Tissue samples were immunoblotted for acetylated (acet.) α-tubulin and total α-tubulin and relative levels determined by densitometry of immunoreactive bands. Values represent the ratio of acetylated α-tubulin to total α-tubulin immunoreactivity. The values in bold highlight those samples with increased ratios present in tissue samples from individuals with chronic alcohol exposure.

Abbreviation: ac., acetylated.

The patient samples we examined were not from a curated set but rather what has come available to us through the Live On Nebraska organ procurement program provided by organ donors and from a small sample set from the Liver Tissue Cell Distribution System (Minneapolis, MN). As such, the patient data set does not contain proper sex distribution across samples. In an effort to address this limitation, we examined livers from ethanol-fed female mice. Immunoblots of whole homogenates from 3 sets of pair-fed livers showed enhanced acetylated α-tubulin immunoreactivity in samples from ethanol-fed mice (Figure S1, http://links.lww.com/HC9/A217). When quantitated and normalized to total α-tubulin, the acetylated α-tubulin levels were increased 2.6-fold (Figure S1, http://links.lww.com/HC9/A217), consistent with values observed from ethanol-fed male livers.

To confirm that acetaldehyde adduction was also specific to alcohol-induced liver injury, we immunoblotted the human tissue for MAA adducts. The normal liver showed little reactivity, whereas the livers with alcohol-associated injury showed enhanced reactivity (Figure 3C). Notably, a 55 kDa band had increased immunoreactivity in alcohol-exposed livers. For comparison, the same samples were blotted for total α-tubulin (Figure 3C, right panel). Both species showed similar migration patterns consistent with ethanol-induced α-tubulin adduction. Together, these results indicate that our findings are clinically relevant and further confirm that the protein modifications are enhanced in alcohol-induced liver injury.

### αTAT1 overexpression induces microtubule acetylation, whereas acetaldehyde addition induces adduction

**FIGURE 4:**
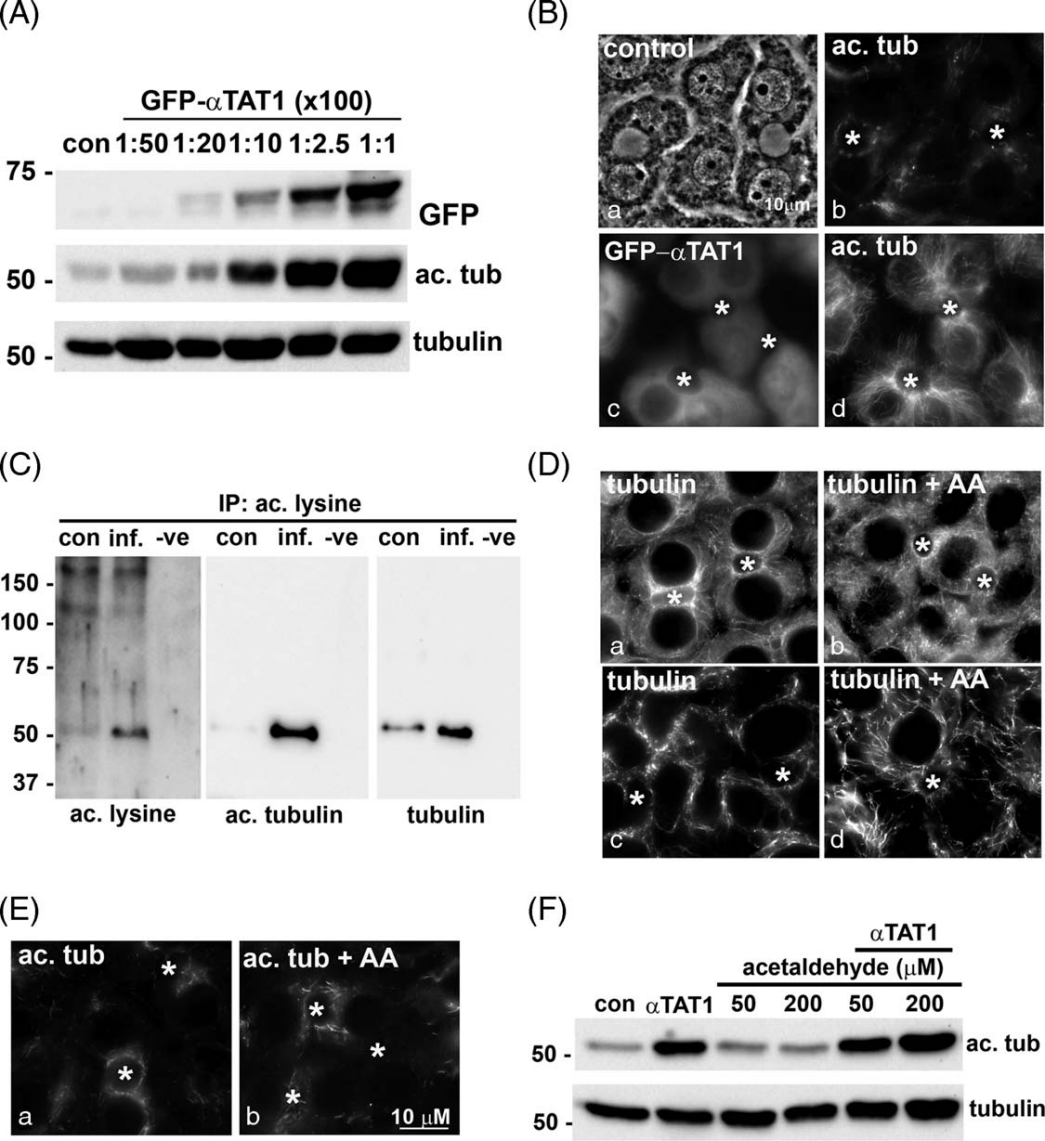
αTAT1 expression specifically promotes tubulin acetylation, whereas acetaldehyde addition promotes tubulin acetaldehyde adduction. (A) WIF-B cells were infected with increasing amounts of recombinant GFP-αTAT1 adenovirus for 24 hours. Whole lysates from uninfected (con) cells or cells overexpressing GFP-αTAT1 were immunoblotted for GFP-αTAT1 (using anti-GFP antibodies), acetylated, or total α-tubulin, as indicated. Virus dilutions indicated are 100-fold greater than that used and range from 1:5000 to 1:100. Molecular weight standards are indicated on the left in kilodalton. (B) WIF-B cells were infected with GFP-αTAT1 (1:200 dilution) for 24 hours (c and d). Control (uninfected) and expressing cells were immunolabeled for acetylated tubulin (b and d). GFP labeling for αTAT1 is shown in c. Asterisks mark bile canaliculi. (C) Total protein (1 mg) from control (con) or GFP-αTAT1–infected cells (inf) was immunoprecipitated (IP) using acetylated lysine affinity beads. The negative control (−ve) represents an IP in the absence of lysate. The bound fraction was immunoblotted for acetylated lysine (to detect all acetylated proteins, left panel) or for acetylated α-tubulin (middle panel) or total α-tubulin (right panel). Molecular weight standards are indicated on the left in kilodalton. (D) WIF-B cells were treated in the absence or presence of 200 μM acetaldehyde (AA) for 24 hours and immunostained for α-tubulin. The cells in panels c and d were prepermeabilized before fixation to remove the soluble pool of tubulin for enhanced visualization of the intact microtubule network. In E, cells were immunolabled for acetylated tubulin. Asterisks mark bile canaliculi. (F) WIF-B cells were treated with increasing concentrations of acetaldehyde in the absence or presence of GFP-αTAT1 expression for 24 hours, as indicated. Whole lysates were prepared and immunoblotted for acetylated and total α-tubulin. Molecular weight standards are indicated on the left in kilodalton.

To induce the tubulin acetylation in the absence of ethanol, we exogenously expressed the tubulin-specific acetyltransferase, αTAT1 (GFP-αTAT1), using recombinant adenovirus (see Supporting Materials for details, http://links.lww.com/HC9/A217). Immunoblots of lysates from cells infected with increasing virus amounts revealed a dose-dependent increase in GFP-αTAT1 expression, which corresponded to increased acetylated tubulin (Figure 4A). When normalized to total α-tubulin, acetylation was enhanced to nearly 20-fold at the highest amount used. Because ethanol induces microtubule acetylation 2- to 3-fold *in vitro* and *in vivo*, we infected cells with dilutions to induce equivalent acetylation levels. In control cells, the faint labeling of acetylated microtubules was observed near the canalicular surfaces (Figure 4B). In contrast, robust labeling was observed in GFP-αTAT1–expressing cells that were not observed in adjacent uninfected cells (Figure 4B, compare c and d).

To confirm that tubulin is the sole substrate for αTAT1 in WIF-B cells, we immunoisolated the total population of lysine-acetylated proteins from control and GFP-αTAT1–expressing cell lysates using antiacetyl lysine affinity beads and immunoblotted the bound fractions with pan antiacetylated lysine antibodies. In control cells, a faint 55 kDa immunoreactive species was detected with enhanced detection in lysates from GFP-αTAT1–expressing cells (Figure 4C, left panel), consistent with acetylated tubulin. This was confirmed by blotting the bound fractions for acetylated and total tubulin directly (Figure 4C, middle and right panels, respectively). Together, these results indicate that αTAT1 specifically acetylates tubulin.

To promote acetaldehyde adduction, we treated WIF-B cells with acetaldehyde. As a readout for microtubule modification, we examined microtubule morphology. In control cells, microtubules emanate from centrosomal structures located near the apical surfaces (Figure 4D). In acetaldehyde-treated cells, this orientation was maintained, but the microtubules were shorter, more discrete, and more gnarled (Figure 4D, b), as we have described for microtubules in ethanol-treated cells.^18^ This morphological change was more apparent in cells prepermeabilized to rid of soluble tubulin (Figure 4D, c and d). We also ruled out that acetylation was enhanced in cells treated with acetaldehyde to avoid the confounding results. In both control and acetaldehyde-treated cells, few acetylated microtubules were detected (Figure 4E). Immunoblotting confirmed these results. No enhanced tubulin acetylation was observed in cells treated with 50 or 200 μM acetaldehyde, whereas robust labeling was seen with cells expressing αTAT1 (Figure 4F). In addition, no enhanced acetylation was observed in GFP-αTAT1–expressing cells that were also treated with acetaldehyde (Figure 4F). Together, these results confirm the validity of our approach.

### αTAT1 overexpression or acetaldehyde addition impairs clathrin-mediated internalization and microtubule-dependent protein trafficking

**FIGURE 5:**
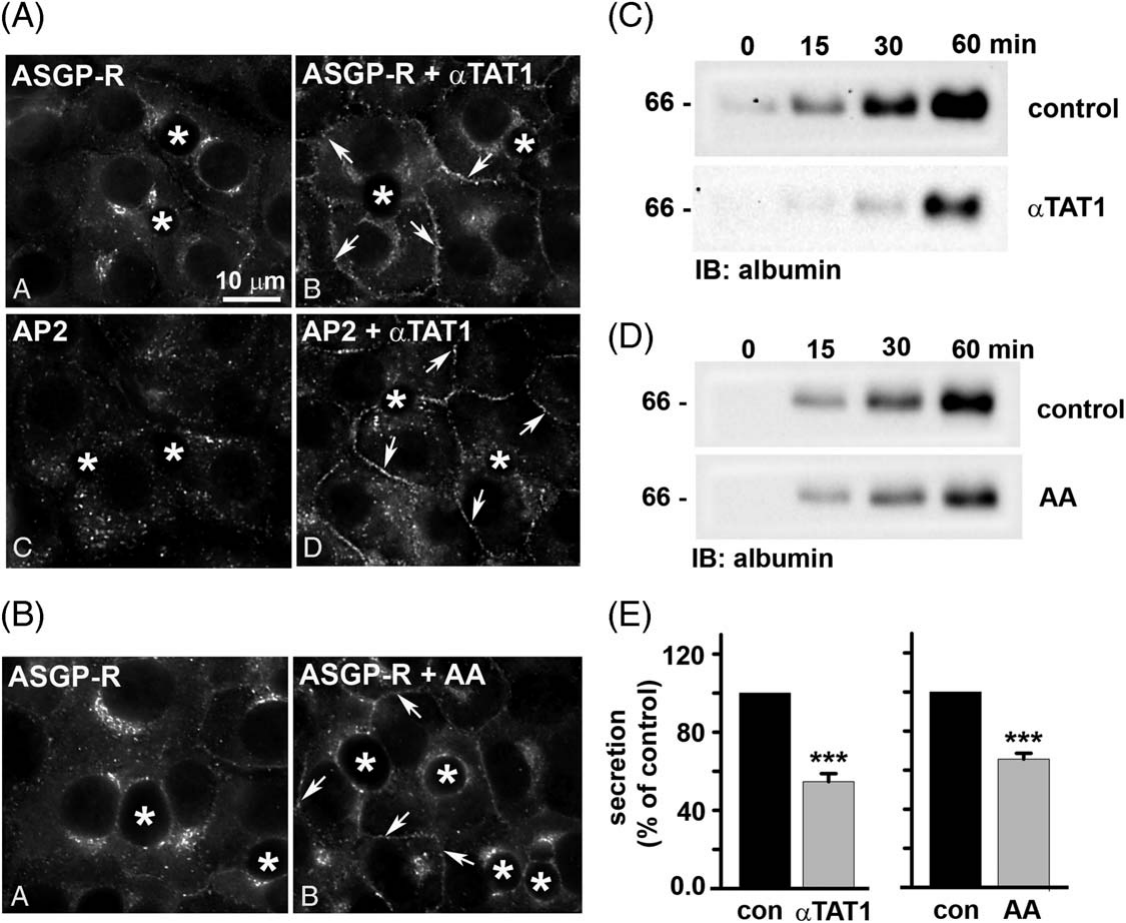
αTAT1 expression or acetaldehyde addition impair clathrin-mediated internalization and secretion. (A and C) WIF-B cells were infected with recombinant adenovirus expressing GFP-αTAT1 (1:200 dilution) for 24 hours. (B and D) WIF-B cells were treated with 200 μM acetaldehyde (AA) for 24 h. Cells in A and B were immunolabeled for ASGP-R or AP2 as indicated. Asterisks indicate bile canaliculi. Arrows point to ASGP-R-positive puncta at the basolateral membrane. Bar=10 μm. In C and D, control (uninfected or untreated) cells or cells expressing GFP-αTAT1 or treated with acetaldehyde (AA) were washed in PBS and reincubated in a serum-free medium. At 0, 15, 30, and 60 minutes after reincubation, aliquots of media were collected and immunoblotted for albumin. Molecular weight standards are indicated on the left in kilodalton. For E, densitometric analysis of the immunoreactive species in C and D was performed, and albumin secretion was plotted as a percentage of control at 60 minutes. Values are expressed as the average±SEM from at least 3 independent experiments. ***p≤0.001. Abbreviations: ASGP-R, asialoglycoprotein receptor.

To determine whether microtubule modifications impair clathrin-mediated internalization, we monitored asialoglycoprotein receptor (ASGP-R) distributions. This receptor is constitutively internalized by clathrin-mediated endocytosis and recycles between the basolateral membrane and early endosomes in the absence or presence of a ligand. In control cells, the majority of ASGP-R was detected in juxtanuclear puncta corresponding to early endosomes (Figure 5A and B, a). In both GFP-αTAT1–expressing and acetaldehyde-treated cells, a striking increase in ASGP-R labeling at or near the basolateral membrane was observed to similar extents (Figure 5A and B, b). Such a shift in distribution is consistent with impaired receptor internalization that was observed in ethanol-treated cells.^25^,^26^,^34^ For comparison, we labeled GFP-αTAT1–expressing cells for adaptor protein (AP2), a known plasma membrane clathrin adaptor protein. In control cells, intracellular punctate AP2 distributions were observed, whereas enhanced basolateral labeling was detected in GFP-αTAT1–expressing cells, also consistent with impaired clathrin-mediated internalization (Figure 5A, c and d).

To assess whether microtubule acetylation and/or adduction impair plus-end–directed motility, we monitored albumin release into the culture medium, as described.^25^ Immunoblots of aliquots at the indicated times revealed that albumin was robustly secreted into the medium and detected within 15 minutes (Figure 5C and D, top panels). However, there was a marked decrease in albumin detection at all times in GFP-αTAT1–expressing and acetaldehyde-treated cells (Figure 5C and D, bottom panels). When quantitated and normalized to total cellular α-tubulin (see Figure S2, http://links.lww.com/HC9/A217, for α-tubulin immunoblots), albumin secretion after 60 minutes was impaired by 45% in GFP-αTAT1–expressing cells and by 35% in acetaldehyde-treated cells (Figure 5E). These values are similar to what we observed in ethanol-treated cells, where secretion was impaired by 55%.^25^,^35^

**FIGURE 6:**
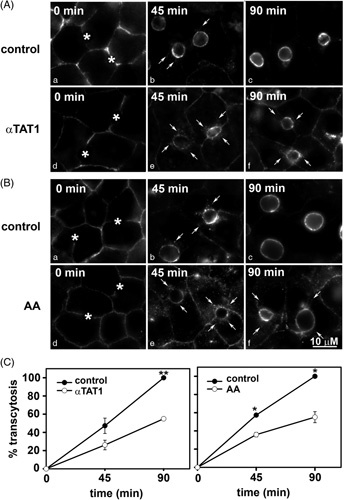
αTAT1 expression or acetaldehyde addition impairs basolateral-to-apical transcytosis. (A and B) Control cells or cells expressing GFP-αTAT1 or treated with acetaldehyde were basolaterally labeled with antibodies specific for the extracellular epitope aminopeptidase N (APN) at 4°C. After excess antibodies were washed away, antibody-antigen complexes were chased for 0, 45, or 90 minutes at 37°C as indicated. Cells were fixed, permeabilized, and labeled with secondary antibodies to detect the transcytosed APN. Asterisks mark bile canaliculi at 0 minutes chase (a and d). Arrows indicate subapically accumulated transcytosing proteins in cells expressing αTAT1 or treated with acetaldehyde (AA). Images are representative of at least 3 experiments. (C and D) Random fields, as shown in A and B, were visualized by indirect immunofluorescence and digitized. From micrographs, the average pixel intensity of each marker at selected regions of interest placed at the apical or basolateral membrane of the same WIF-B cell was measured. The averaged background pixel intensity was subtracted from each value, and the ratio of apical-to-basolateral fluorescence intensity was calculated. Values are plotted as the percent of control and are expressed as the mean±SEM from at least 3 independent experiments. *p≤0.05, **p≤0.01.

To assess minus-end–directed trafficking, we monitored transcytosis of basolaterally labeled APN after 0, 45, and 90 minutes of chase, as described.^22^,^32^ Live cells were basolaterally labeled with antibodies specific to external APN epitopes for 20 minutes at 4°C. The cells were rewarmed to 37°C, the antibody-antigen complexes were chased to the apical surface for either 45 or 90 minutes, and the cells were fixed and processed for indirect immunofluorescence detection of the trafficked proteins. Because tight junctions restrict antibody access to the canalicular surface, only basolateral labeling was detected after a 0-minute chase in all cases (Figure 6A, B). Clear apical labeling was observed in control cells after 45 and 90 minutes of chase (Figure 6A, B, b and c), indicating canalicular delivery. In contrast, significantly decreased canalicular labeling was observed in GFP-αTAT1–expressing or acetaldehyde-treated cells, with a reciprocal increase in intracellular labeling on subcanalicular structures and at the basolateral membrane (Figure 6A, B, e and f). As for the ethanol-treated cells, we propose the subapical structures represent stalled transcytosing vesicles along the modified microtubules, whereas basolateral labeling represents impaired APN clathrin-mediated internalization.^22^ To quantitate transcytosis, we calculated the ratio of canalicular to basolateral fluorescence intensity, as described.^22^ APN transcytosis was impaired by 45% in cells expressing GFP-αTAT1 or treated with acetaldehyde (Figure 6C). These values are remarkably similar to that observed in ethanol-treated cells, where 50% impairment was observed.^22^ Together, these results indicate that either modification alone can impair protein trafficking, as observed in ethanol-treated cells.

### The effects of tubulin acetaldehyde adduction or acetylation are not dose dependent or additive

**FIGURE 7:**
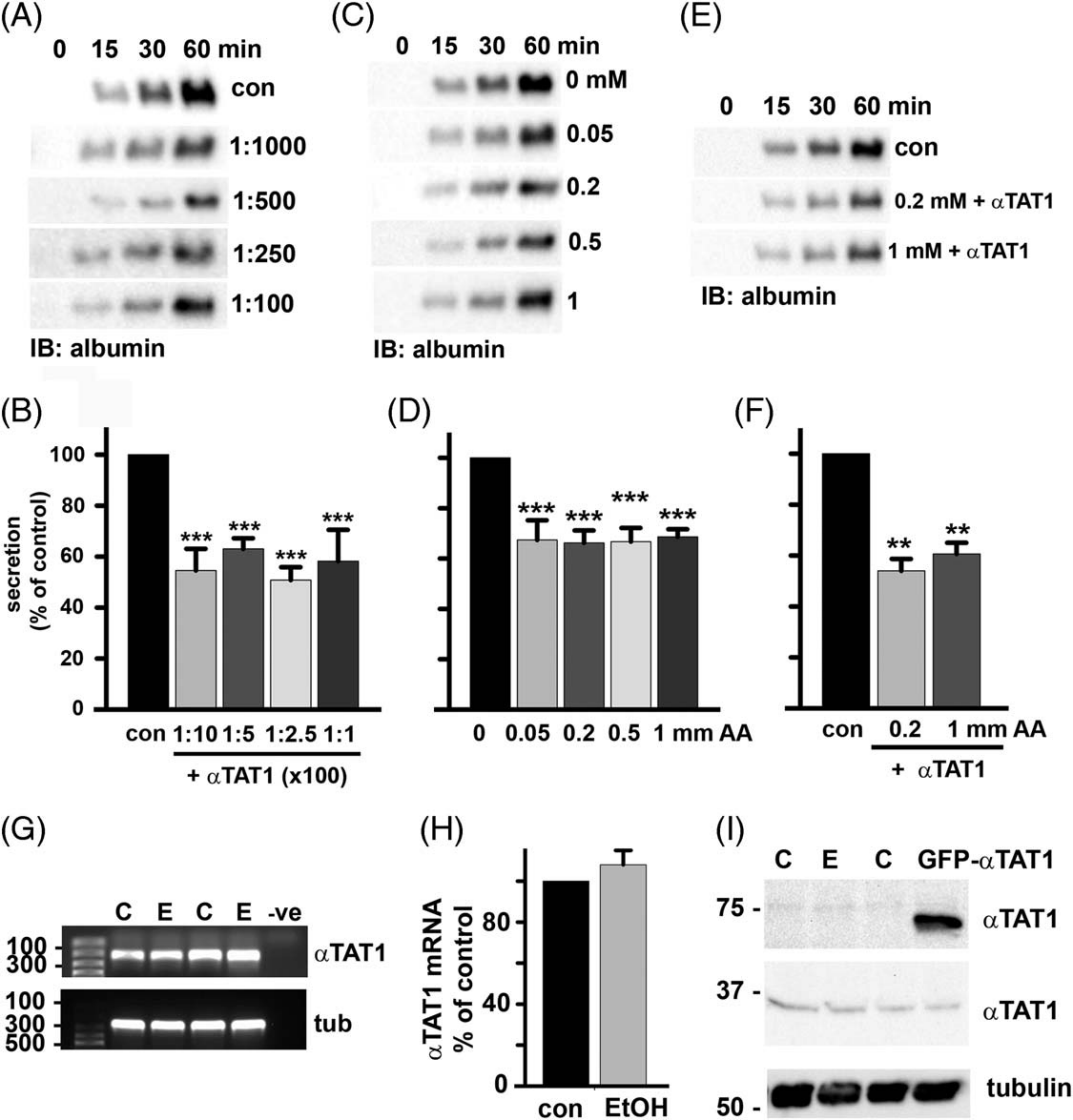
The effects of microtubule modification by acetaldehyde and acetylation on secretion are not dose dependent or additive, nor is αTAT1 overexpressed in ethanol-treated cells. Albumin secretion was monitored as described in Figure 5 in cells treated with increasing amounts of GFP-αTAT1 (A), increasing concentrations of acetaldehyde (C), or in cells overexpressing αTAT1 in the additional presence of 0.2 or 1 mM acetaldehyde (E). Densitometric analysis of the immunoreactive species was performed, and albumin secretion plotted as a percentage of control at 60 minutes for cells infected with increasing amounts of GFP-αTAT1 (B), increasing concentrations of acetaldehyde (D) or in cells expressing GFP-αTAT1 in the additional presence of 0.2 or 1 mM acetaldehyde (F). Values are expressed as the average±SEM from at least 3 independent experiments. **p≤0.01, ***p≤0.001. (G). Agarose gels are shown of αTAT1 (upper panel) and α-tubulin (lower panel) cDNA amplified by reverse-transcription PCR from 1 µg total RNA isolated from cells treated in the absence or presence of 50 mM ethanol for 72 hours. Size standards are indicated on the left in base pairs. (H) αTAT1 mRNA expression levels were normalized to α-tubulin expression levels by densitometry and plotted as the percentage of control. (I) Whole cell lysates from control, ethanol-treated, and GFP-αTAT1-expressing cells were immunoblotted for endogenous αTAT1. Molecular weight standards are indicated on the left in kilodalton. Values are expressed as the mean±SEM from at least 3 independent experiments.

We next asked whether microtubule adduction or acetylation showed dose-dependent responses. Somewhat surprisingly, secretion was impaired to the same extent in cells infected with increasing virus amounts (Figure 7A). For all concentrations, secretion was impaired by 45% (Figure 7B and see Figure S2, http://links.lww.com/HC9/A217, for α-tubulin immunoblots used as a loading control). Similarly, in cells treated with increasing concentrations of acetaldehyde, no dose dependence was observed, with all concentrations displaying 35% impairment (Figure 7C, D). Interestingly, a dose response was not observed at lower acetaldehyde concentrations, but rather a threshold response was observed. At 0.5 or 5 μM acetaldehyde, no decrease in secretion was observed (Figure S3A, http://links.lww.com/HC9/A217). However, an abrupt decrease was observed at 10 and 25 μM (Figure S3, http://links.lww.com/HC9/A217). When quantitated and normalized to total cellular α-tubulin, secretion was impaired ~40% (Figure S3B, http://links.lww.com/HC9/A217), as was observed in cells treated with higher acetaldehyde concentrations (Figure 7D). Because both acetylation and adduction target lysine residues, we also tested whether the modifications additively impair secretion. For these experiments, the cells were infected with virus amounts that promote modest acetylation in the presence of 200 μM or 1 mM acetaldehyde. Immunoblots revealed no additive effects in albumin secretion (Figure 7E); secretion was impaired by ~40% for both (Figure 7F).

### αTAT1 is not overexpressed in ethanol-treated cells

Although we overexpressed αTAT1 as a means to promote microtubule acetylation in the absence of ethanol, our results prompted us to ask whether its levels were increased in ethanol-treated cells. We examined endogenous αTAT1 mRNA levels by semiquantitative reverse-transcription PCR (see Table S4 for details, http://links.lww.com/HC9/A217). The results from 2 independent experiments are shown where no apparent changes in αTAT1 levels were observed (Figure 7G), which was confirmed upon quantification (Figure 7H). Total endogenous αTAT1 protein levels also did not change in ethanol-treated cells or cells overexpressing GFP-αTAT1 (Figure 7I). Thus, alcohol-induced αTAT1 expression does not explain increased tubulin acetylation, consistent with our findings that decreased histone deacetylase 6 (HDAC6) microtubule association may explain enhanced acetylation.^36^

## DISCUSSION

We began our analysis by confirming that microtubule acetylation and adduction are present in the livers from ethanol-exposed mice/rats and extended our studies to show that they are also present in livers from individuals exhibiting alcohol-induced injury. We further demonstrated that the modifications may also be more specific to alcohol-induced injury. We also determined that microtubule acetylation or acetaldehyde adduction impaired both plus-end (secretion) and minus–end-directed (transcytosis) microtubule-dependent protein trafficking and clathrin-mediated internalization to the same extent as seen in ethanol-treated cells. The modifications showed no dose dependence alone, nor did they have additive effects. Finally, we found that ethanol does not lead to increased αTAT1 levels, thereby ruling out the possibility that enhanced αTAT1 expression explains alcohol-induced protein acetylation.

### Tubulin acetylation or adduction alone impairs protein trafficking to the same extent as ethanol

We found that tubulin acetylation and adduction led to remarkably similar levels of impaired vesicle delivery as observed in ethanol-treated cells. In addition, the modifications showed no dose dependence, and they were not additive. This suggests that substoichiometric tubulin modifications lead to maximal impairment in protein trafficking just as substoichiometric tubulin adduction by acetaldehyde led to impaired polymer assembly.^17^ This further suggests that only a few critical lysines need to be modified to confer impaired vesicle motility. At present, little is known about the steady-state stoichiometries of the 2 modifications in ethanol-treated cells. Tubulin acetylation is apparently substoichiometric *in vivo*, as its levels can be increased 2- to 3-fold by ethanol exposure. However, this increase is saturable and does not increase with increased ethanol exposure or by increased ethanol concentrations,^18^ yet αTAT1 expression led to acetylation levels 20-fold over a steady state. Thus, strict mechanisms must be in place to maintain steady-state modification levels in control and ethanol-treated cells. It will be important to determine the specific stoichiometries of each modification to better understand the cellular phenotypes and pathologies associated with modified microtubules.

Another open question is to what extent acetaldehyde adduction and acetylation “compete” for these critical lysines. From our preliminary proteomics analysis of purified tubulin from ethanol-treated cells, we determined that the acetylation and adduction can occur on the same lysine (eg, on lysine 40) and that peptides from the same sample of alcohol-exposed tubulins had either no modifications at lysine 40 or were acetylated or were adducted (unpublished data). This suggests that the modifications can compete for the same lysine, but who wins *in vivo* is not understood, nor are their relative stabilities known as microtubules undergo dynamic instability and repolymerization. Finally, the addition of CYP2E1 inhibitors or anti-oxidants does not alter microtubule acetylation or protein trafficking in ethanol-exposed hepatic cells,^35^ indicating that the other modifications of oxidative stress are not contributing to the phenotypes observed.

### Alcohol-induced microtubule acetylation and acetaldehyde adduction are clinically relevant modifications

We determined that tubulin acetylation was significantly enhanced in humans with alcohol-induced liver fibrosis and cirrhosis (by 4.3-fold), which is consistent with the enhanced acetylation observed in livers from ethanol-fed mice/rats and WIF-B cells.^18^ Interestingly, patients with NAFLD showed little increased acetylation, whereas nonalcohol-associated fibrosis livers had no enhanced acetylation. Although we acknowledge the limitations of our human sample set with respect to sex distribution or disease etiologies, we plan to expand the human sample analysis as additional donor livers are procured or from other small sample sets. Our finding that both male and female rodents exhibit alcohol-induced tubulin modifications suggests that both male and female human samples will exhibit similar responses. The ultimate goal is to examine whether alcohol exposure specifically promotes tubulin acetylation and whether the defects in protein trafficking that are promoted by tubulin acetylation are also specific to alcohol-exposed hepatocytes. Such information may explain differences in the complications observed from different forms of liver injury and inform clinicians of specific treatment strategies.

In addition to tubulin, the acetylation of numerous proteins is known to be enhanced by ethanol treatment, and in some cases, the modifications have been linked to altered hepatic function.^7^ Many naturally occurring and synthetic KDAC agonists and KAT antagonists are well tolerated in humans and are in various stages of clinical trials for the treatment of cardiovascular and neurodegenerative diseases, inflammation, and metabolic disorders.^5^ In general, these pharmacological agents are targeted to nuclear KDACs or KATS, thereby leading to changes in gene expression. Because alcohol induces the acetylation of a host of mitochondrial and cytosolic proteins,^6^,^21^ the identification of agonists to the major cytoplasmic (HDAC6) or mitochondrial (SirT3, 4 and 5) deacetylases is needed. Importantly, such nonnuclear targeted agents may reduce protein acetylation without altering gene expression, thereby potentially reducing side effects. Nonetheless, our results suggest that changing the cellular acetylation levels or scavenging free aldehydes are feasible approaches to treating alcohol-associated liver disease.

## Supplementary Material
