# Supplementary material for: Alcohol-induced tubulin post-translational modifications directly alter hepatic protein trafficking
Source: Hepatol Commun. 2023 Mar 24;7(4):e0103. doi: 10.1097/HC9.0000000000000103 (PMC10043593; doi:10.1097/HC9.0000000000000103)
Supplement: Supplementary file 1 [file hc9-7-e0103-s001.docx]

**Alcohol-induced tubulin post-translational modifications directly alter**

**hepatic protein trafficking**

Raghabendra Adhikari^1^, Ramyajit Mitra^1^,Robert G. Bennett^2, 3, 4^,

Benita L. McVicker^2, 3^ and Pamela L. Tuma^1^

^1^Department of Biology, The Catholic University of America, Washington, D.C., USA

^2^Research Service, VA Nebraska-Western Iowa Health Care System, Omaha, NE, USA

^3^Department of Internal Medicine, University of Nebraska Medical Center, Omaha, NE, USA

^4^Department of Biochemistry and Molecular Biology, University of Nebraska Medical Center, Omaha, NE, USA

**CORRESPONDENCE:**

Pamela L. Tuma, PhD

Department of Biology

The Catholic University of America

620 Michigan Avenue, NE

103 McCort-Ward

Washington, DC 20064

[tuma@cua.edu](mailto:tuma@cua.edu)

**Materials and Methods**

**Animal studies**

Male 6-week old C57BL/6J mice (Jackson Laboratories, Bar Harbor, ME) were treated with CCl_4_ (diluted 1:7 in sunflower oil) and injected intraperitoneally twice per week with a dose of 1 μl/g body weight (0.125 μl/g CCl_4_) for 4 or 12 weeks (6 mice per group). Control mice received injections of oil alone. Mice had free access to water and chow throughout the study. Mice were euthanized 24 h after the final injection, and blood and tissues collected. All procedures were approved by the Nebraska-Western Iowa Health Care System Institutional Animal Care and Use Committee. Serum levels of alanine transaminase (ALT), aspartate transaminase (AST), alkaline phosphatase (ALP) and albumin were determined using an automated clinical chemistry analyzer. Hydroxyproline content was measured using the Quickzyme collagen kit (Cedarlane, Burlington, Canada). Values are expressed as μg hydroxyproline per gram liver wet weight. Formalin-fixed paraffin-embedded sections were stained with picrosirius red to monitor collagen deposition (1). Images were captured using a Nikon Eclipse 80i microscope and DSQilMc digital camera (Boyce Scientific, Inc., Gray Summit, MO). Staining intensity was quantified with histomorphometry using ImageJ software (National Institutes of Health, Bethesda, MD) using image deconvolution and threshold functions (2, 3). At least 10 non-overlapping fields per section were analyzed. Female, 8-week-old mice (Charles River, Wilmington, MA) were weight-matched and pair-fed Lieber DeCarli control or ethanol liquid diets for four weeks. All procedures were approved by the Washington DC Veterans Affairs Medical Center Institutional Animal Care and Use Committee.

**Virus production and infection**

GFP-αTAT1 plasmids were purchased from Addgene (Cambridge, MA) (pEF5B-FRT-GFP-TAT1, no. 27099, a gift from M. Nachury) and cloned for recombinant adenovirus production using the Invitrogen Gateway System (Thermo Fisher Scientific). The ViraPower Adenoviral Expression System (Thermo Fisher Scientific) was used to generate recombinant a GFP-αTAT1 adenoviruses. WIF-B cells were infected with recombinant adenovirus particles for 60 min at 37°C as described (4). Complete medium was added to the cells and they were incubated an additional 16–24 h to allow for protein expression.

**Statistical analysis**

Results are expressed as the mean ± SEM (n=6). Comparisons between experimental groups were made using the Student’s two-tailed *t* test for paired data.  One-way ANOVA was used when more than two groups were analyzed, followed by Holm-Sidak multiple pairwise comparison. *p* values ≤ 0.05 were considered significant.

**Supporting Table S1. Antibodies used in the study**

| **Antibody name** | **Catalog number** | **Manufacturer** | **Technique** | **Dilution/Quantity** |
| --- | --- | --- | --- | --- |
| α-tubulin | T9026 (C-DM1A) | Sigma–Aldrich (St. Louis, MO) | Immunofluorescence | 1:400 |
|  |  |  | Western blot | 1:7500 |
| acetylated α-tubulin | T6793 (C-6-11B-1) | Sigma–Aldrich | Immunofluorescence | 1:250 |
|  |  |  | Western blot | 1:2000 |
| C6ORF134 (mec17 or αTAT1) | SAB2100308 | Sigma–Aldrich | Western blot | 1:2000 |
|  |  |  |  |  |
| clathrin adaptor protein 2 (AP2) | NB600-1545 (C- AP6) | Novus Biologicals (Centennial, CO) | Immunofluorescence | 1:100 |
| acetylated-lysine residues | 9441S | Cell Signaling (Danvers, MA) | Immunofluorescence | 1:1000 |
| α-tubulin | 2125S | Cell Signaling | Western blot | 1:2500 |
| acetyl-lysine affinity beads | AAC04 | Cytoskeleton Inc., Denver, CO | Immunoprecipitation | 50 μL |
| GFP | 66002 (C-1E10H7) | Proteintech (Rosemont, IL) | Western blot | 1:7000 |
| aminopeptidase N (APN) | Kindly provided by Dr. Ann Hubbard (5) | | Antibody trafficking | 1:100 |
| asialoglycoprotein receptor (ASGP-R) | Kindly provided by Dr. Ann Hubbard (6) | | Immunofluorescence | 1:100 |
| rat serum albumin (RSA) | Kindly provided by Dr. Ann Hubbard (7) | | Western blot | 1:10000 |
| malondialdehyde-acetaldehyde (MAA) | Kindly provided by Dr. Geoffrey M. Thiele (8) | | Immunofluorescence | 1:100 |
|  |  |  | Western blot | 1:1000 |
| goat anti-rabbit IgG peroxidase | A6154 | Sigma–Aldrich | Western blot | 1:10000 |
| goat anti-mouse IgG-HRP | A4416 | Sigma–Aldrich | Western blot | 1:10000 |
| goat anti-guinea pig IgG-HRP | A7289 | Sigma–Aldrich | Western blot | 1:10000 |
| Alexa Fluor 568 goat anti-rabbit IgG | A11036 | Thermo Fisher Scientific (Waltham, MA) | Immunofluorescence | 1:400 |
| Alexa Fluor 488 goat anti-rabbit IgG | A11034 | Thermo Fisher Scientific | Immunofluorescence | 1:400 |
| Alexa Fluor 568 goat anti-mouse IgG | A11031 | Thermo Fisher Scientific | Immunofluorescence | 1:400 |
| Alexa Fluor 488 goat anti-mouse IgG | A11029 | Thermo Fisher Scientific | Immunofluorescence | 1:400 |

**Supporting Table S2. CCl_4_-treated mice exhibit significant tissue injury.**

| **Liver injury marker** | | **4 wk control** | **4 wk + CCl_4_** | **12 wk + CCl_4_** |
| --- | --- | --- | --- | --- |
| ALT (U/L) | 73.95 ± 12.88 | | 7396.87 ± 1842.24** | 4800.00 ± 1785.08* |
| AST (U/L) | 335.98 ± 207.05 | | 2918.15 ± 492.25* | 2375.00 ± 985.80* |
| ALP (U/L) | 118.15 ± 9.48 | | 138.25 ± 4.46* | 103.00 ± 4.07* |
| Albumin (g/dL) | 12.34 ± 0.12 | | 13.09 ± 0.16** | 13.70 ± 0.15*** |
| Hydroxproline (μg/g tissue) | 0.23 ± 0.03 | | 0.42 ± 0.06** | 0.39 ± 0.02* |
| Sirius red | 0.95 ± 0.12 | | 5.65 ± 0.43*** | 7.82 ± 0.38*** |
| (% total area) |  |  |  |  |

The numbers represent mean ± SEM (n = 6). **p* ≤ 0.05, ***p* ≤ 0.01, ****p* ≤ 0.001 (CCl_4_ compared to control). Hydroxyproline content is expressed μg hydroxyproline per gram liver wet weight. Abbreviations: ALP, alkaline phosphatase; ALT, alanine aminotransferase; AST, aspartate aminotransferase; CCl_4_, carbon tetrachloride; wk, weeks

**Supporting Table S3. Demographic and clinical data for healthy controls and individuals with NAFLD or alcohol-associated liver disease (samples used for immunoblotting)**

| **Liver pathology** | **Age**  **(yrs)** | **Gender** | **BMI** | **ALT**  **(U/L)** | **AST**  **(U/L)** | **ALP**  **(U/L)** | **Albumin**  **(g/dL)** | **Bilirubin (mg/dL)** | **MELD score** |
| --- | --- | --- | --- | --- | --- | --- | --- | --- | --- |
| **normal 1** | 50 | M | NA | NA | NA | NA | NA | NA | NA |
| **normal 2** | 55 | F | NA | NA | NA | NA | NA | NA | NA |
| **normal 3** | NA | NA | NA | NA | NA | NA | NA | NA | NA |
| **normal 4** | 59 | M | NA | NA | NA | NA | NA | NA | NA |
| **normal 5** | 67 | F | NA | NA | NA | NA | NA | NA | NA |
| **normal 6** | 34 | M | NA | NA | NA | NA | NA | NA | NA |
| **normal 7** | 38 | F | 24.9 | 65 | 77 | 85 | 2.8 | 0.8 | 6 |
| **NAFLD 1** | 57 | F | 27.6 | 378 | 357 | 413 | 2.2 | 0.4 | 6 |
| **NAFLD 2** | 59 | M | 24.2 | 46 | 22 | 31 | 2.2 | 0.6 | 6 |
| **NAFLD 3** | NA | NA | NA | NA | NA | NA | NA | NA | NA |
| **NAFLD 4** | 53 | M | NA | 60 | NA | 236 | 3.1 | 25.1 | 24 |
| **NAFLD 5** | 43 | M | 44.0 | 88 | 60 | NA | NA | 9.2 | 27 |
| **NAFLD 6** | 67 | F | NA | 44 | 32 | 98 | 3.0 | 8.8 | 40 |
| **fibrosis** | 6 | F | 20.2 | 45 | 46 | 104 | 2.2 | NA | NA |
| **fibrosis/alcohol**  **incomplete cirrhosis** | 50 | M | 37.7 | 15 | 12 | 59 | 3.0 | 0.7 | 11 |
| **cirrhosis/alcohol 1** | 51 | M | 27.7 | 24 | 30 | 62 | 5.1 | 10.1 | 25 |
| **cirrhosis/alcohol 2** | 71 | M | 29.0 | 50 | 31 | NA | NA | 3.2 | 22 |
| **cirrhosis/alcohol 3** | 64 | M | 29.0 | 50 | 31 | NA | NA | 3.2 | 22 |
| **cirrhosis/alcohol 4** | 50 | M | 25.7 | 72 | 31 | NA | NA | 5.3 | 23 |
| **cirrhosis/alcohol 5** | 32 | M | 18.4 | 45 | 23 | NA | NA | 12.9 | NA |

Human normal livers 1-6 and NAFLD livers 3-6 were obtained through the Liver Tissue Cell Distribution System, Minneapolis, Minnesota, which was funded by NIH contract # HSN276201200017C. All other livers were from Live On Nebraska organ recovery program. NA, not available

**Supporting Table S4. Primer sequences used for RT-PCR**

| **Molecule** | **Forward Primer** | **Reverse Primer** |
| --- | --- | --- |
| **Rat α-TAT1** | 5’-CCAGTATATGTTACAGAAAGAGCGA-3’ | 5’-AGCACGAGAGTGTCGAGTTG-3’ |
| **Rat α-tubulin** | 5’-CCAGGGCTTCTTGGTTTTCC-3’ | 5’-CGCTCAATGTCGAGGTTTCT-3’ |

Total RNA was extracted from 1 x 10^6^ cells control or treated cells using the RNeasy mini kit (Qiagen). Samples were eluted with 50 μl of RNase-free water. RT-PCR was performed with 1 μM RNA-template using OneStep RT-PCR kits (Qiagen). PCR was performed using the following parameters: 30 min at 50^o^C, 15 min at 95^o^C, 30 cycles of 30 s at 94^o^C, 30 s at 55^o^C, 1 min at 72^o^C, and 10 min at 72^o^C. PCR products were analyzed on 1% agarose gels. Rat α-tubulin was used as a loading control. The relative levels of the amplified αTAT1 fragments (normalized to tubulin) were determined by densitometric analysis using ImageJ.


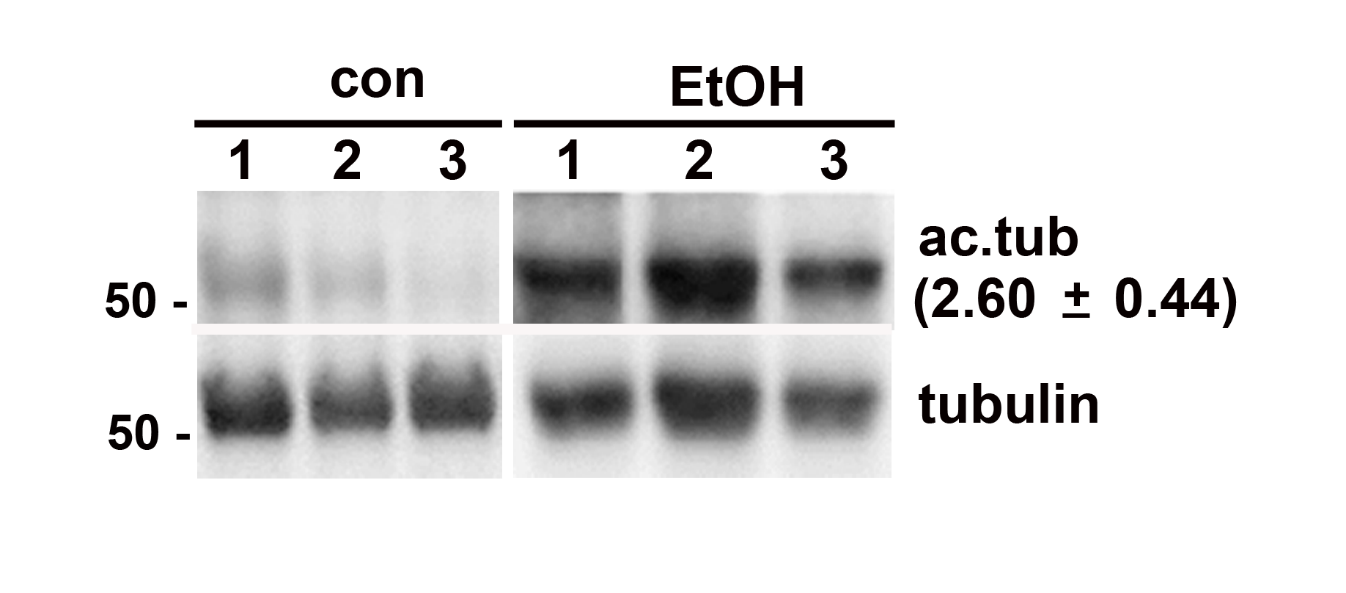


**Supporting Figure S1.** Ethanol exposure induces tubulin acetylation in female mice. Liver homogenates from three sets of pair-fed (control, C; ethanol, E) female mice were immunoblotted for total or acetylated α-tubulin. Relative levels of tubulin acetylation were determined by densitometry and normalized to total α-tubulin. The fold-increase in acetylated tubulin in ethanol-treated cells is indicated in parentheses and represents the mean ± SEM from the three pairs of mice.

**
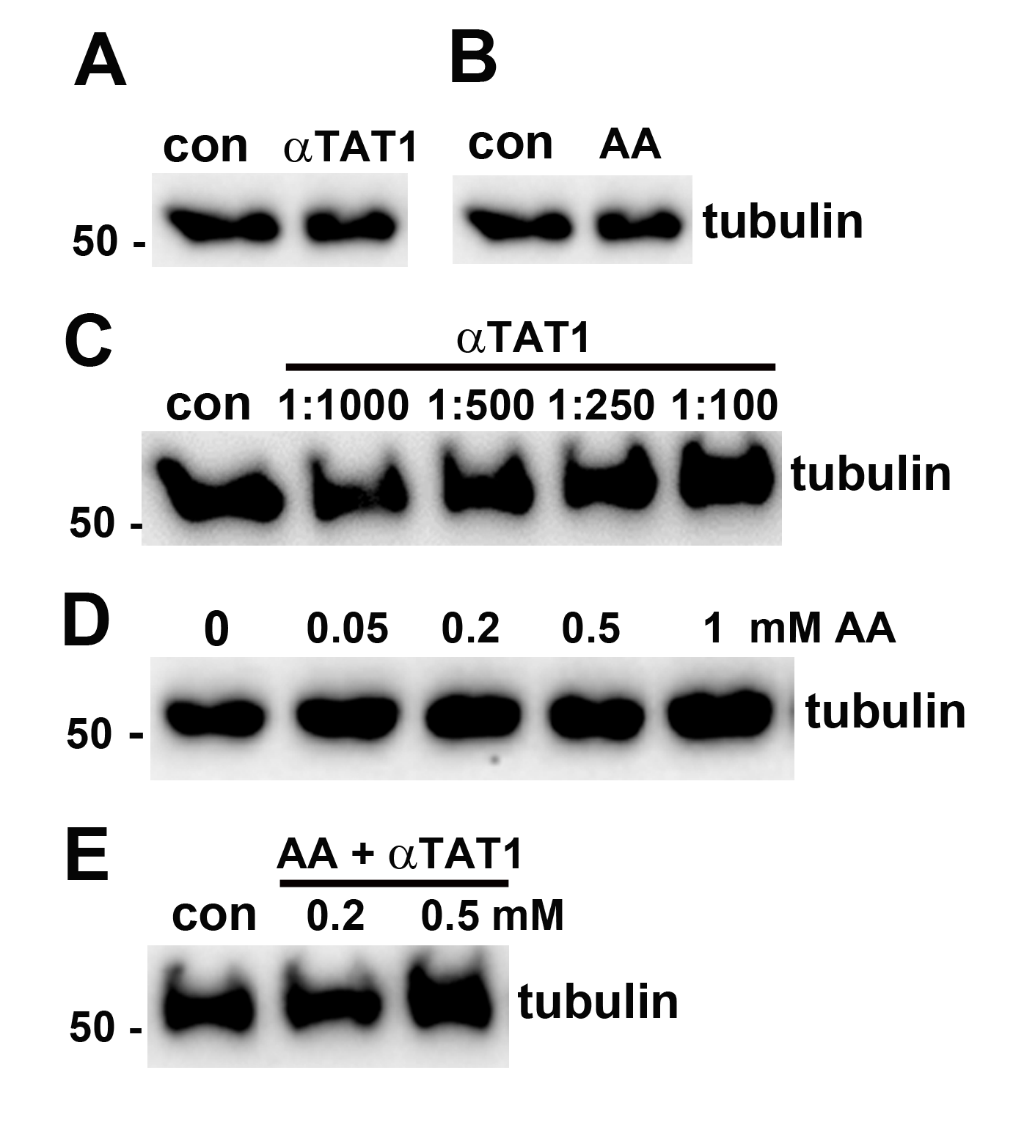
**

**Supporting Figure S2.** Loading controls for albumin secretion experiments. Cell lysates from (A) control or αTAT1 overexpressing cells from Figure 5C, (B) acetaldehyde (AA)-treated cells from Figure 5D, (C) control or αTAT1 overexpressing cells from 7A, (D) control or acetaldehyde-treated cells shown from Figure 7C and (E) control, αTAT1 overexpressing and/or acetaldehyde-treated cells from Figure 7E were immunoblotted for total α-tubulin to serve as a loading control.

**
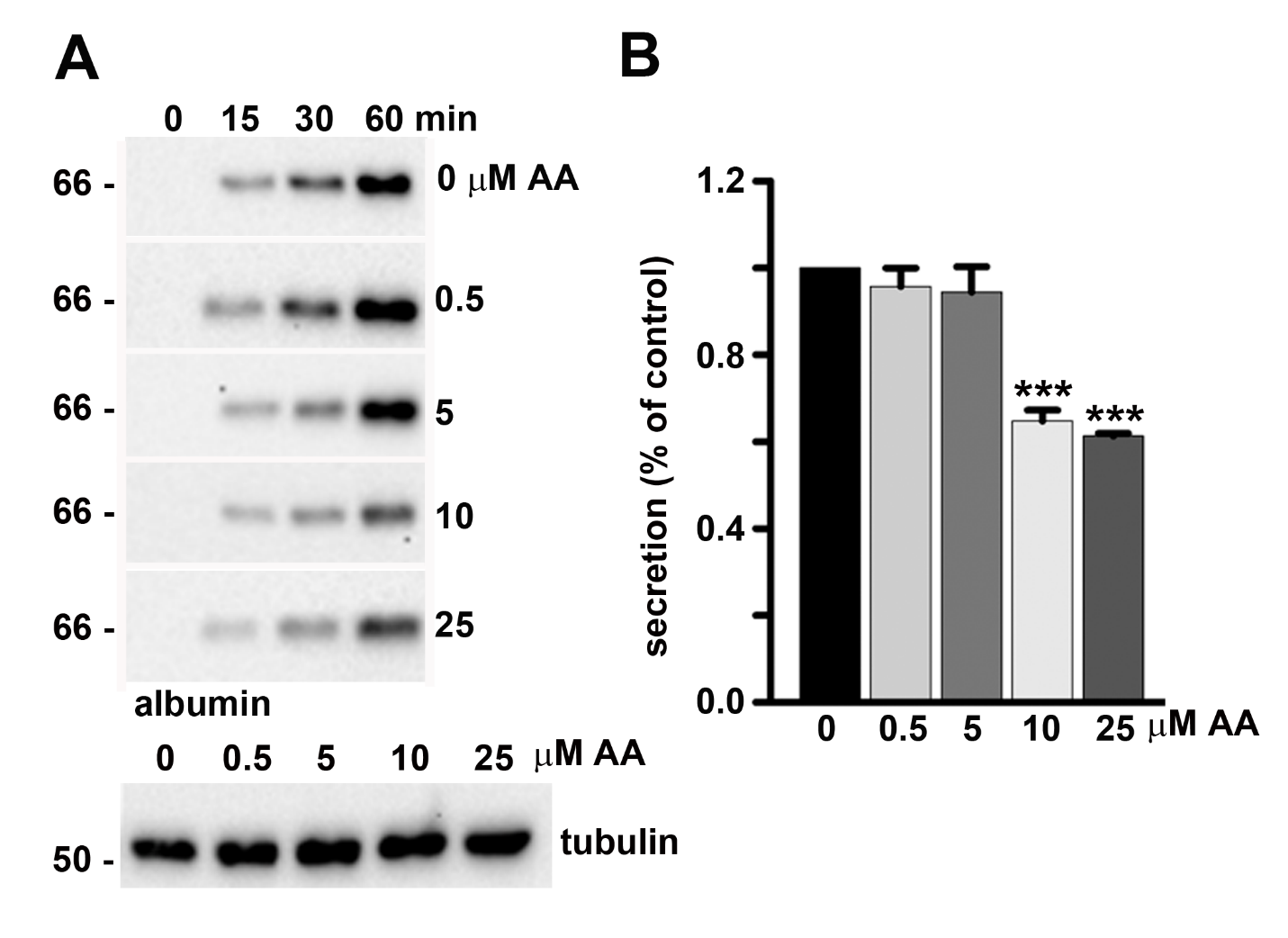
**

**Supporting Figure S3.** The effects of microtubule modification by acetaldehyde on secretion are not dose dependent at low concentrations. (A) WIF-B cells were treated with increasing concentration of acetaldehyde (AA) for 24 h. The cells were washed in PBS and re-incubated in serum-free medium.  At 0, 15, 30, and 60 min after reincubation, aliquots of media were collected and immunoblotted for albumin (upper panel). Cell lysates from each condition tested were immunoblotted for total α-tubulin to serve as a loading control (lower panel). Molecular weight markers are indicated on the left in kDa. (B) Densitometric analysis of the immunoreactive species in A was performed and albumin secretion plotted as a percentage of control at 60 min normalized to total α-tubulin levels.  Values are expressed as the average ± SEM from at least three independent experiments. ****p* ≤ 0.001.

**References**

1. Junqueira LC, Bignolas G, Brentani RR. Picrosirius staining plus polarization microscopy, a specific method for collagen detection in tissue sections. Histochem J. 1979;11:447-455.

2. Collins TJ. ImageJ for microscopy. Biotechniques. 2007;43 25-30.

3. Ruifrok AC, Johnston DA. Quantification of histochemical staining by color deconvolution. Anal Quant Cytol Histol. 2001;23:291-299.

4. Bastaki M, Braiterman LT, Johns DC, Chen YH, Hubbard AL. Absence of direct delivery for single transmembrane apical proteins or their "Secretory" forms in polarized hepatic cells. Mol Biol Cell 2002;13:225-237.

5. Ihrke G, Neufeld EB, Meads T, Shanks MR, Cassio D, Laurent M, Schroer TA, et al. WIF-B cells: an in vitro model for studies of hepatocyte polarity. J Cell Biol 1993;123:1761-1775.

6. Bartles JR, Braiterman LT, Hubbard AL. Endogenous and exogenous domain markers of the rat hepatocyte plasma membrane. Journal of Cell Biology 1985;100:1126-1138.

7. Griffo G, Hamon-Benais C, Angrand PO, Fox M, West L, Lecoq O, Povey S, et al. HNF4 and HNF1 as well as a panel of hepatic functions are extinguished and reexpressed in parallel in chromosomally reduced rat hepatoma-human fibroblast hybrids. J Cell Biol 1993;121:887-898.

8. Thiele GM, Klassen LW, Tuma DJ. Formation and immunological properties of aldehyde-derived protein adducts following alcohol consumption. Methods Mol Biol 2008;447:235-257.
